# A Case of Peritonitis, with Fœcal Abscess, in Which the Patient Recovered; with Remarks

**Published:** 1834-01-01

**Authors:** John Burne


### *A Case of Peritonitis, with Fœcal Abscess, in which the Patient recovered; with Remarks.* By JOHN BURNE, M.D.

THOMAS TUBMAN, aged thirty-five, a tailor, lusty, rather bloated, and in the habit of drinking ale freely, was seized, in November, 1832, after breakfast, with sharp griping pain in the umbilical region, which in the afternoon had become so severe as to require medical aid. Advice was accordingly procured, and medicine administered, which was immediately rejected, as was everything he took. The case being regarded as one of colic, calomel, opium, castor oil, and aromatic confections, were administered, but without avail.

On the third day, as he was now a patient of the Dispensary, I visited him, and found the disease to be a well-marked peritonitis. He was lying on his back in bed, quite still, with the knees drawn up, complaining of constant pain in the middle of the abdomen, of urgent vomiting, and constipation. The abdomen was full, had a solid doughy feel, and was preternaturally hot; the pain was aggravated by slight pressure; there was no tumour in the usual situations of hernia; the respiration was rather frequent; the pulse contracted; the tongue thickly furred and of a dingy white; with a general febrile heat on the surface of the body. Anxiety was depicted on the countenance; he spoke of being very low, and muscular tremor was noticed while feeling the pulse.

Sanguis e brachio ad ℥x. detrahatur. ℞. Hydr. Submur. gr. xxiv.; Opii, gr. iv., fiant pilul. xij. sumat unam secundâ quâque horâ. Empl. Cantharidis amplum abdomini imponatur.

These measures were followed on the next (the fourth)

day by some abatement in the urgency of the symptoms; but, as the bowels had not been acted upon, he was directed to take the following medicines: ℞. Sodæ Tartarizatæ, ʒi.; Sodæ Carb. ℈i.; Acidi Tartar. gr. xv. M. fiat pulv. tertiâ quâque horâ ex aquâ in impetu ipso effervescentiæ bibendus donec alvus satis soluta fuerit.

The aperient had the desired effect of producing plentiful evacuations, and the gums had now (the fifth day) become sore from the mercury. The violence of the symptoms was considerably subdued, yet the stomach remained irritable, the abdomen tender and painful, the patient low, and the strength much exhausted. Here the amendment, which had been progressive and satisfactory, ceased in a great degree, and the next five or six days were passed in a state of apprehension, the local symptoms persisting, the organic functions continuing to be disturbed, and his strength to decline seriously for want of support, which the foul tongue and irritable stomach refused.

Although, from the tenth to the twentieth day, some improvement had taken place, the state of the patient was nevertheless still precarious. About this period there was discovered a circumscribed tumour of considerable size below and about the navel: it was deep seated, being manifestly in the abdominal cavity; the other part of the belly having become soft and natural. The patient now experienced a severe rigor, and the tumour day after day increased, approached the surface, and presently gave evidence of an obscure fluctuation. As the abdominal symptoms were concentrated more and more in the tumour, so the sympathetic disturbance of the constitution diminished, and the patient, being able to take nourishment, his powers began to return. The integuments soon became inflamed, fluctuation was distinct, and an incision being made into the abscess, there issued a large quantity of thin, dark, fœtid matter, and sulphuretted hydrogen gas.

The operation was followed by great relief: the general health improved daily, the tumour diminished, the discharge lost its offensive character by degrees, and at the last was merely a thin serous fluid. The abscess healed in the course of a fortnight, and the patient recovered perfectly.

The first point of interest in this case is the character of the pain at the onset, which caused the disease to be regarded as colic. The character of the pain was exacerbating, and was described by the patient himself as griping; and so far it was certainly more allied to the pain of colic than of peritonitis; but the error in diagnosis arose from allowing the judgment to be formed by the pain alone, whereas, had an opinion been drawn from the symptoms collectively, no doubt of the disease being inflammation could have existed. The still position of the patient on the back; the tenderness and solid fulness of the abdomen, in addition to the pain; the sympathy of the stomach; the frequent pulse, and general febrile movement, stamped the inflammatory character of the disease beyond all question, and formed a striking contrast to the constant change of position, the quiet pulse, and the absence of febrile movement, in colic.

The second point of interest is the continuance of the abdominal symptoms, and of the general disturbance of the system, after the period when, in cases of idiopathic peritonitis, they usually subside; and this in despite of the remedial measures, and of the influence of mercury, which had been pushed to salivation. The reason, however, is explained by the eventual formation of the fœcal abscess.

The leading facts of the case, taken collectively, go far to prove that it was a peritonitis excited by an ulcerative perforation of some part of the small intestine, and the consequent escape of the contents of the bowel through the perforation into the peritoneal cavity. The circumscribed character of the tumour and of the abscess shew that the peritonitis was circumscribed also; that the effused fœcal matter had been walled-in by the inflammation, and eventually eliminated by the natural method of an abscess.

The same facts shew that the perforation of the intestine was very minute, and that the quantity of matter which escaped from the bowel was not considerable; for, had the perforation been extensive, and the quantity of matter effused large, the peritonitis would have been diffuse as well as vehement, and, as in all such cases, quickly fatal.

Peritonitis, arising from ulcerative perforation of some part of the alimentary canal, is not unfrequent. So long as an ulcer is confined to the mucous and submucous tissues, it is not productive of serious consequences; but, when it has destroyed the muscular tunic, the corresponding part of the peritoneal tunic soon dies and sloughs; the perforation is then completed, the contents of the alimentary canal escape into the peritoneal cavity, and excite an immediate inflammation, manifested by the sudden and rapid development of all the signs peculiar to peritonitis.

In order that these cases may be treated successfully, (provided they are such as admit of recovery,) it is of the utmost consequence that their true nature should be at once discovered; the principle of treatment applicable to them differing widely from that which is applicable to an idiopathic peritonitis.

An idiopathic peritonitis is to be treated on the simple principle of subduing inflammation: inflammation being the sum and substance of the disease. But, in peritonitis symptomatic of ulcerative perforation of the intestine, the cause of the inflammation, the effused matter, continues in operation, and can only be removed by the tedious formation of an abscess. The principle, then, on which such a symptomatic inflammation should be treated must be consistent with the process which nature has to accomplish. The indication of cure is to moderate and control the violence of the inflammation, and at the same time to husband the powers of the system, in order that it may be enabled to endure the formation of an abscess, which is required for the elimination of the offending matter. Had the patient in question been subjected to great depletion, he would have sunk long before the natural process of cure could have been accomplished.

